# Integrating stakeholder perspectives into the translation of cell-free fetal DNA testing for aneuploidy

**DOI:** 10.1186/gm348

**Published:** 2012-06-21

**Authors:** Lauren C Sayres, Megan Allyse, Mildred K Cho

**Affiliations:** 1 Stanford Center for Biomedical Ethics, Stanford University, 1215 Welch Road Modular A, Stanford, California, 94301, USA; 2 Department of Pediatrics, Division of Genetics, Stanford University, 1215 Welch Road Modular A, Stanford, California, 94301, USA

## Abstract

**Background:**

The translation of novel genomic technologies from bench to bedside enjoins the comprehensive consideration of the perspectives of all stakeholders who stand to influence, or be influenced by, the translational course. Non-invasive prenatal aneuploidy testing that utilizes cell-free fetal DNA (cffDNA) circulating in maternal blood is one example of an innovative technology that promises significant benefits for its intended end users; however, it is currently uncertain whether it will achieve widespread clinical implementation. We conducted qualitative interviews with 18 diverse stakeholders in this domain, including prospective users of the technology and healthcare personnel, researchers and developers, and experts in social, legal, and regulatory aspects of genetic technology, and a pilot survey of 62 obstetric healthcare providers. Analysis of interview and survey data was combined with a review of the proceedings of a full-day, multidisciplinary conference on the topic and published scientific and ethics literature surrounding this and other relevant technologies.

**Discussion:**

We constructed potential pathways for technological implementation, identified broad stakeholder classes party to these translational processes, and performed a preliminary assessment of the viewpoints and interrelations among these diverse stakeholders. Some of the stakeholders whose priorities are critical to understand and integrate into translation include pregnant women and their families; healthcare providers; scientists, their institutions or companies, and the funding agencies that support them; regulatory and judicial bodies; third-party payers; professional societies; educational systems; disability rights communities; and other representatives from civil society. Stakeholder interviews, survey findings, and conference proceedings add complexity to these envisioned pathways and also demonstrate a paramount need to incorporate an iterative stakeholder analysis early and throughout the translational endeavor. We believe that the translational framework that we have developed will help guide crucial future stakeholder mapping and engagement activities for cffDNA aneuploidy testing and inform novel methods of technology assessment for other developments in the growing field of genomic medicine.

**Summary:**

Mapping potential pathways for implementation and exploring the attitudes and interrelations of diverse stakeholders may lead to more effective translation of a novel method of prenatal aneuploidy testing.

## Background

The 2011 vision of the National Human Genome Research Institute aspires to fulfill the ultimate goal of the Human Genome Project by applying the results of genomic research to diverse areas of healthcare [1]. However, this vision appears to be based on the assumption that translation is primarily a matter of greater understanding of biology and development of genomic technology. Instead, we suggest that successful genomic translation also requires the design of translational pathways that take into account the objectives and values of a wide range of stakeholders. Because translation of medical technologies rarely proceeds down a path of inevitable advances, we propose that translation is more likely when all stakeholder perspectives are deeply integrated into each phase of the research, development, implementation, and policy-making process [2-8]. To be successful, these pathways must additionally be mindful of broader context: the existence of related technologies, social dynamics and stakeholder convictions about these technologies, and existing political and economic frameworks for translation and use. As evidenced by the thwarted translation of other promising genetic technologies, such as genetically modified crops and gene transfer technologies, a failure to explore contextual elements in advance of translation may result in unfulfilled stakeholder expectations, stakeholder frustration and resistance, and ultimately, translational failure, even after a new biotechnology has been made commercially available [9-11].

By exploring potential translational pathways in the context of one nascent application of genetic technology - prenatal aneuploidy testing using cell-free fetal DNA (cffDNA) - we illustrate how stakeholder perspectives may have significant and direct effects on the course of translation. We hope that the development of this framework of translational pathways and stakeholder interactions provides impetus for a more textured evaluation of the influences and interests of diverse stakeholders and continuous engagement of these actors in implementing cffDNA technology. Furthermore, we believe that this broad methodology for technology assessment - moving beyond evaluation of the primary characteristics of a technology towards a comprehensive integration of stakeholder values and contextual elements - may be applied to other genomic technologies with unique sets of stakeholders and divergent translational pathways.

### Tracing translational pathways for cffDNA testing for aneuploidy

Several recent studies have demonstrated that cffDNA in maternal blood can be used for the non-invasive prenatal detection of aneuploidy [12-16]. Because this testing requires only a maternal blood draw - eliminating any risk of miscarriage - and can be performed earlier in pregnancy than existing tests, cffDNA testing has the potential to make aneuploidy testing routine for all pregnancies. Several companies have announced plans to commercialize cffDNA testing for aneuploidy, including trisomy 13, 18, and 21 (Down syndrome), and three companies have already started to offer trisomy testing in the United States [17-20]. Although substantial media attention has been directed towards the introduction of these tests, it is not clear whether the development of this technology will lead to the immediate and widespread adoption that some observers predict [21].

This analysis has been conducted to explore the underlying values of diverse actors in the research, development, and use of cffDNA technology in order to guide the translational enterprise [22-24]. As the first stage in this assessment, we interviewed 18 stakeholders with diverse interests in this realm to sketch the landscape of the technology and its ethical, legal, and social context and to understand how various stakeholders conceptualize translation. Stakeholders were identified as representatives of previously described parties to genetic technology translation, including prospective technology adopters, various healthcare personnel, academic researchers, commercial developers, community activists, and experts on law and regulation in this arena, through citation in the academic literature and media and via snowball sampling [4,24,25]. Semi-structured interviews were conducted, lasting approximately one hour each. We also conducted a pilot survey of 62 healthcare providers in attendance at a Continuing Medical Education conference on advances in obstetrics and gynecology; the findings from this survey have been published elsewhere but are provided as important references for this analysis [26]. Our Institutional Review Board approved both the series of interviews and survey, and all interview and survey subjects provided consent to participate. We also reviewed the transcripts of a full-day, multi-disciplinary conference, 'The Coming Revolution in Prenatal Genetic Testing? Scientific, Ethical, Social, and Policy Responses to Maternal Serum Cell-free Fetal DNA Testing', jointly hosted by the Stanford University School of Medicine and Stanford Law School in May 2010, and pertinent academic literature on cffDNA testing, related technologies, and surrounding ethical, legal, and social implications [16].

## Discussion

### Results

Throughout data collection and review, we began to narrow in on six classes of relevant stakeholders while tracing how the technology might be translated from bench to bedside. Iterative feedback from colleagues knowledgeable about the technology confirmed that our framework was a legitimate and useful tool for identifying stakeholders and assessing their influences and interrelations in the translational context. The findings of our interviews, survey, and the multi-disciplinary conference add nuance to many of the translational complexities and contingencies set forth by the National Human Genome Research Institute's vision and inform the specific translational pathways that we describe. These data suggest that a wide range of scientific and social factors, including characteristics of the existing system of prenatal care and testing but beyond simply the technical characteristics of cffDNA testing, may affect implementation.

**Figure 1:**
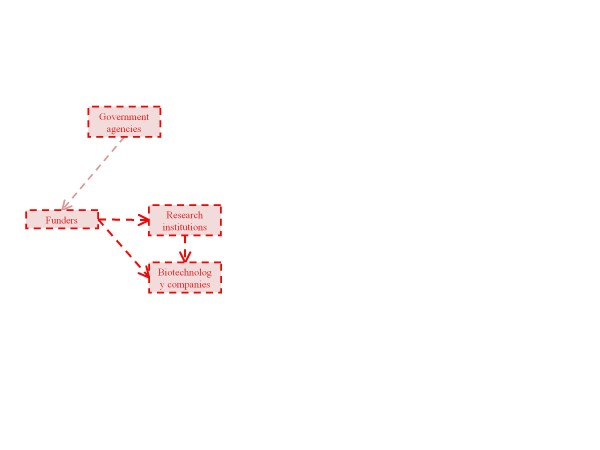
**The allocation of resources to research and development is a crucial factor in technology development**. Thick dashed arrows represent potential pathways for the flow of technology, resources, and knowledge among primary groups of stakeholders. Thin dashed arrows represent interactions and influences among broader groups of stakeholders. The stakeholders and pathways highlighted in red will be the focus of each section.

**Figure 2:**
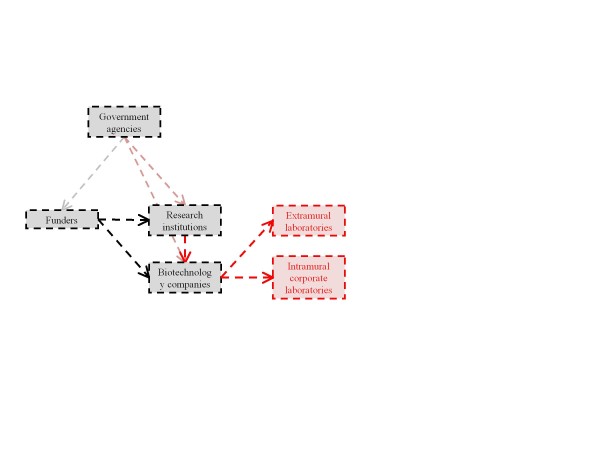
**The structure and location of research and development activities has downstream effects on translation**.

**Figure 3:**
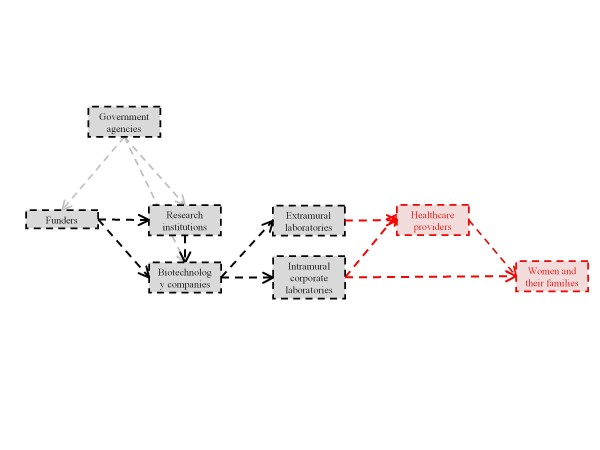
**The route of provision of testing raises significant ethical and logistical questions**.

**Figure 4:**
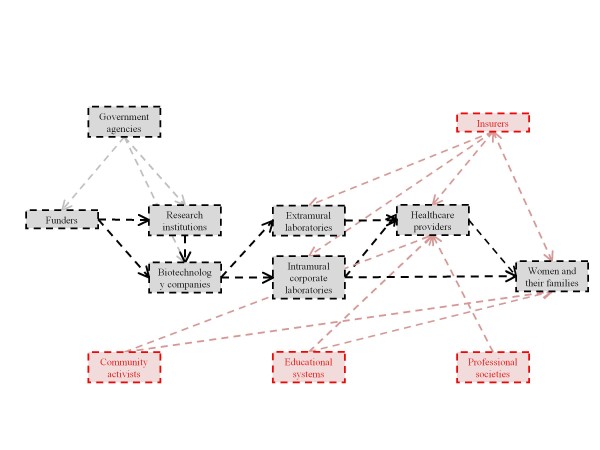
**Provider and patient uptake of new testing may be affected by a variety of external groups**.

**Figure 5:**
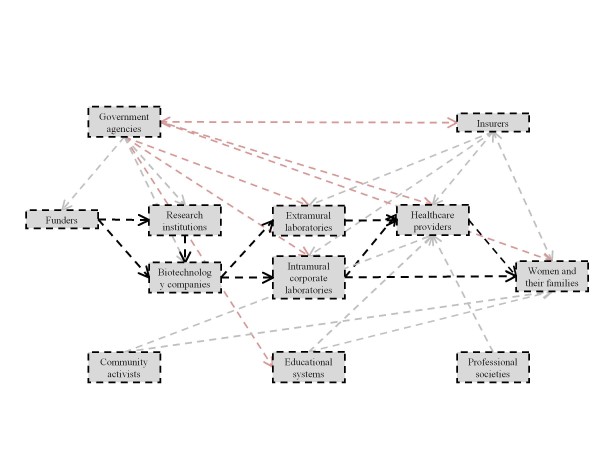
**Legal, regulatory, and funding decisions by government agencies will change the testing landscape**.

**Figure 6:**
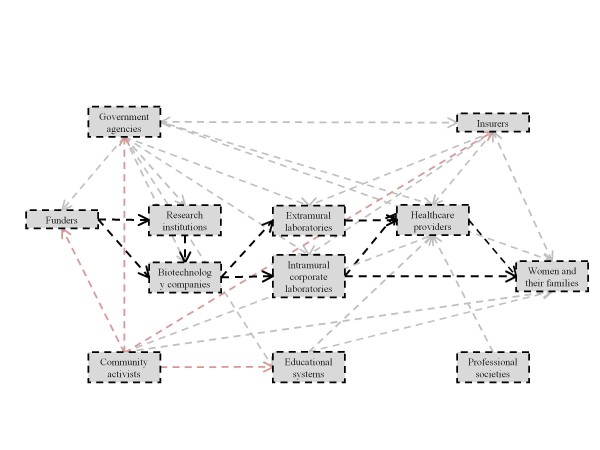
**Activists can place pressure on key stakeholders, which may directly affect translation**.

In the following sections, organized by our six identified stakeholder classes, we sketch potential translational pathways for cffDNA testing for aneuploidy, including their points of divergence, and the individuals and institutions that influence, and may be influenced by, their progress. Increasingly complex diagrams of these pathways (Figures 1 to 6) are presented as additional sets of stakeholder interactions are explored. These pathways are not mutually exclusive but are delineated in order to highlight their salient features and ethical, legal, and social consequences for stakeholders. Where appropriate, concrete policy recommendations are presented.

### Resource allocation

Funding for the research, development, and initial application of a technology is necessary to any translational endeavor (Figure 1). The allocation of resources for the translation of cffDNA testing demands sufficient expectation that the current uncertainties regarding the promised value of this technology will resolve favorably [27]. Additionally, mobilization of all resources - capital, equipment, and personnel - will need to be scaled up throughout the progression of implementation until it is clear whether or not the technology can, and will, be applied in a widespread manner.

Thus far, the majority of fiscal support for the research phase of cffDNA testing, which is occurring primarily in academic research centers, has been in the form of grants or endowments from government agencies, private foundations, and institutional funds. The biotechnology industry has subsequently been pursuing the development of scalable tests based upon research results using venture capital, grants, and other investments [28-30]. Until revenue can be secured, however, additional resources are required to apply the technology in its intended use. Biotechnology firms that are conducting studies to evaluate the clinical application of cffDNA testing must be capable of attracting sufficient financial support to underwrite these activities. As cffDNA testing for aneuploidy becomes clinically available, the source and size of capital investment will need to adjust to meet the changing priorities of its commercializers and users [31]. Private and state health insurance agencies have already become involved in dictating the implementation of this technology at the point of care, through decisions of whether to cover the first commercially available cffDNA tests for aneuploidy [32]. Given insurers' continued support for extant prenatal screening and diagnostic techniques, it is essential to understand the priorities of these funders in order to identify likely trajectories for the translation of this new technology. Statements made by our study's interviewees suggest that, where possible, insurance coverage should be decided on the basis of several inputs, including the wishes of insurance program enrollees to receive testing, medical standard of care, and comprehensive comparative analyses to existing technologies.

### Research and development

Scientists at academic institutions have designed several methods to test cffDNA for aneuploidy, and the biotechnology industry is currently aiming to validate these methods through clinical trials (Figure 2) [16]. During our stakeholder interviews, several scientists claimed that reliable testing is already feasible in the presence of skilled personnel and unlimited resources, which is consistent with the growing body of literature [12-15]. However, in considering the scaling-up or expansion of such testing to broader populations and resource-limited settings, developers face the challenge of maintaining sufficient levels of reliability to compete with existing, highly reliable diagnostic tests. A similar technology, which attempted to use fetal cells circulating in maternal blood for prenatal diagnosis, showed some success on a small scale, but a large trial failed to demonstrate sufficiently robust results, resulting in the almost complete rejection of the technology [33,34]. Initial evidence for cffDNA testing for aneuploidy indicates that this technology may achieve more promising outcomes; however, ongoing validation and dissemination of study results will be an important facet of successful clinical implementation of this technology [12-15]. In both interviews and the literature, we have witnessed several physician calls for validation studies that are reproducible across populations of all risk levels and variable clinical and laboratory conditions, which were ultimately never achieved by fetal cell technologies [35].

Many researchers in this field hold dual roles as faculty in academic laboratories and as executives, board members, or consultants for biotechnology companies tasked with commercializing cffDNA tests. Several of these individuals have been awarded patents for cffDNA testing strategies, and the licensing and information-sharing strategies of these patent holders have dictated which biotechnology companies are in a position to translate these methods into clinical applications. Thus far, these companies have utilized their in-house laboratories to conduct aneuploidy testing [13,36]. However, once a satisfactory large-scale commercial test has been developed, it is unclear whether companies will require that all samples be sent to their intramural laboratories for testing (and whether they have the infrastructure for such large-scale operations) or whether they will sub-license their methods such that independent clinical laboratories can perform the tests. In previous instances, companies with rights to patents for specific genes have stringently enforced monopolies through the threat of lawsuits and limited the provision of commercial testing to their internal laboratories [37].

If patent holders for cffDNA testing choose to restrict licensing, there may be a significant impact on availability of the technology, raising concerns of distributive justice, in addition to the potentially troubling implications for provider and patient independence in clinical decision-making [38]. Several studies suggest that market exclusivity resulting from patents and restrictive licensing strategies for genetic tests negatively impacts development, cost, and access to testing [39,40]. The ongoing judicial case challenging patents covering the breast and ovarian cancer susceptibility genes *BRCA1 *and *BRCA2*, held by the company Myriad Genetics, may thus have a significant effect on the course and implications of patenting for cffDNA testing if the validity of patents on genes or genetic diagnostic technologies is called into question [41]. Furthermore, because of the possibility of overlap among existing patent claims surrounding cffDNA testing, the enforcement of patents through infringement suits may have a drastic effect on the translational route of this testing. Despite the fact that legislation, such as the Bayh-Dole Act, is intended to promote translation from academic institutions to the biotechnology industry in just this way, enforcement of patents on cffDNA testing may have the unintended consequence of raising barriers to dissemination [42]. While protectable intellectual property is an important and sometimes necessary incentive for product research and development, relevant commercializers and policy-makers should bear in mind the potential broad repercussions of patenting and licensing practices on cffDNA testing for aneuploidy [24].

### Product dissemination

If biotechnology companies intend to conduct cffDNA testing for aneuploidy through commercial laboratories, the possibility of direct-to-consumer (DTC) test provision, rather than provision through a healthcare professional, arises (Figure 3). DTC genetic testing has inspired intense debate as the medical community and the public try to strike a balance between empowering individuals to learn about their genetic makeup and protecting them from information with an unsound scientific basis or unclear health implications [43,44]. The current controversy over the appropriateness of placing genetic information directly in the hands of consumers becomes more pronounced when decisions to continue or terminate a pregnancy may result from inaccurate or misunderstood test results [45]. Responses to our pilot survey of obstetric healthcare providers demonstrate reservation about the potential availability of DTC cffDNA testing, and furthermore, respondents and interviewees alike indicated that they believe that genetic counseling should be utilized in order for pregnant women wishing to receive testing to understand this technology's indications, limitations, and implications [26]. A policy requiring comprehensive consent and counseling procedures before provision of genetic testing would additionally be in line with the positions of several relevant professional societies, including the American College of Medical Genetics, the National Society of Genetic Counselors, and the American Medical Association [46-48].

Existing cffDNA tests for trisomy 21 and RhD blood group type are currently available only via physician referral, whereas non-invasive prenatal sex testing can be ordered directly by a pregnant woman via the internet [16,17]. With the exception of commercial trisomy 21 tests, no data on the uptake of cffDNA tests have been made publicly available yet, so it is unclear how many tests are being performed. In a previous study of obstetric providers, a majority reported moderate to low levels of knowledge about availability of cffDNA testing, although most were simultaneously interested in offering this type of testing for aneuploidy [26].

Most predominant in dictating provider uptake of cffDNA testing may be the recommendations put forward by relevant professional societies, as surveyed healthcare providers demonstrated nearly unanimous reliance on such guidelines in future decision-making surrounding the adoption of cffDNA testing [26]. Historically, rapid and widespread uptake of prenatal alpha-fetoprotein screening for neural tube defects was almost exclusively the result of one statement issued by the legal committee of American Congress of Obstetricians and Gynecologists, despite its marked incompatibility with a previously issued statement from the organization [49]. Moreover, this uptake occurred at a time when clinicians were apprehensive about the limitations of such screening and were still calling for further studies and services coordination before screening was accessed by broad patient populations [49]. Fear of liability may ultimately have driven implementation of this technology. In the case of cffDNA testing for aneuploidy or other indications, professional societies should comprehensively consider the ethical, legal, social, political, and economic repercussions of testing before issuing unified messages for their constituents.

A number of other factors will affect whether physicians do ultimately offer these tests to their patients: whether a physician order is required for testing, the reliability and timing of the test, clinicians' perception of effectiveness and trust in the testing laboratories, insurance coverage, and perceived or expressed patient wishes. Furthermore, the current framework of prenatal screening and diagnostic options for genetic conditions such as aneuploidy will also affect whether providers adopt cffDNA testing; a reluctance to accept novel technologies or a vested interest in existing technologies may present barriers to widespread implementation. Assessing provider values and attitudes surrounding how this testing should be made commercially available will be an essential step in ensuring that the goals and characteristics of testing match the objectives of its stakeholders.

### Product uptake

Like many medical technologies, the interface between end users and cffDNA testing for aneuploidy influences the trajectory of its implementation (Figure 4). Questions about how patients perceive and interpret benefits and risks in their choice to use (or not to use) this testing are critical. Potential benefits may include non-invasiveness, earlier timing, and increased reliability over existing screening tests. Personal reasons to decline cffDNA testing are more difficult to tease apart but may include objections to prenatal testing or selective termination of pregnancy, either for any genetic condition or specifically for a given aneuploidy; as with the refusal of other prenatal testing, these objections may stem from individual moral judgments or experiences, familial, provider, or social pressures, religious traditions, and broader cultural attitudes [50-53]. The diagnostic capability of cffDNA testing for aneuploidy will also inform how patients reflect on the benefits and costs of this technology relative to existing screening and diagnostic tests. Costs and accessibility of prenatal care or the availability of resources to raise a child with a given aneuploidy may additionally influence pregnant women at the time of decision-making.

In addition, access to information about a technology impacts uptake. In the past, DTC sex testing using cffDNA has generated controversy when consumers, who felt that they had received insufficient information about the possibility of inaccurate results, brought a class-action suit against the company offering the test [54]. Negative publicity, and especially legal action, has the potential to modify and even reverse the course of implementation of cffDNA testing. Additionally, this case elucidates the fact that the non-invasive nature and thus modified benefit-risk calculation for cffDNA testing may undermine the integrity of the informed consent process, even when tests are provided solely through healthcare professionals [55]. Given that pregnant patients undergo a number of unrelated blood draws during early pregnancy, there is evidence to suggest that even trained professionals are not always thorough in obtaining informed consent when the physical risks of a test are so significantly reduced [56]. There is a concern that patients, if unaware that they are undergoing prenatal testing, will feel that their autonomy and decision-making has been compromised [57]. In order to avoid a loss of trust in novel prenatal testing methodologies, both healthcare providers - including obstetricians, genetic counselors, specialized nurses, and other allied health professionals - and potential patients need to be educated about the nature and availability of cffDNA testing. Stakeholders with whom we engaged additionally recommended genetic counseling as a necessary step in attaining the full engagement of patients in the decision-making process, which is consistent with our findings in the healthcare provider population [26].

Through coverage decisions, insurance agencies also have the ability to influence how and whether cffDNA testing for aneuploidy is accessed. One company currently offering cffDNA tests for trisomy 21 has set drastically differential prices ($1,900 as opposed to $235) for consumers based on whether they have insurance coverage [17]. Many women may not be able to afford the out-of-pocket expenses or may choose to decline more expensive options when insurers already cover other tests. Insurance programs will likely assess the cost-benefit ratio of this technology in the context of the established framework of prenatal screening and diagnostic tests for aneuploidy. By exploring testing reliability, timing, and indications, insurers will determine whether implementation is appropriate as a reimbursable primary or secondary screening mechanism or as a diagnostic technique. If a decision is made to not cover cffDNA testing for aneuploidy, this testing may remain accessible only to a wealthy few or never realize commercial success at all. Regional mechanisms for offering testing to large subsets of the population, such as the California Prenatal Screening Program, must also be considered in determining the relevant stakeholders in the dissemination of this technology. If state law requires the offering and coverage of cffDNA testing for all pregnant women seeking prenatal care, as California law currently requires for integrated screening, uptake may be significantly increased. If cffDNA testing for aneuploidy is found to be a cost-effective, desirable prenatal technology, federal and state reimbursement and dissemination strategies should strive for equity in access across all appropriate patient populations.

### Legal context

Government agencies will play an important role in the implementation of cffDNA testing for aneuploidy at several translational stages (Figure 5). As a primary source of funding for research on prenatal testing and a potential buyer of this testing (through state insurance agencies), the government's control over financial resources has the potential to drive forward or inhibit development and use of this technology. Judicial decisions also have the potential to influence translational pathways through pertinent legal cases, such as those involving patenting and licensing, consumer interactions with companies, or communications between patients and healthcare providers by means of wrongful birth suits [58,59]. For example, a recent lawsuit in Oregon resulted in the awarding of several million dollars to parents claiming to have otherwise ended a pregnancy had they received an accurate prenatal diagnosis of trisomy 21; with new technologies like cffDNA testing being made available, pressures will mount for physicians to avoid liability by offering any and all tests to ensure that affected pregnancies are detected [60]. Other stakeholders will have important influences over the initiation and outcomes of such legal cases; for example, in the aforementioned case involving Myriad Genetics, physicians, patients, professional societies, and community activists filed a suit that may influence the legality of gene patenting. In the case of cffDNA testing for aneuploidy, we have already started to witness a surge of legal action pursued by various patent and license holders that may critically influence the characteristics of technological implementation [61].

Currently, government regulation of many genetic tests does not extend beyond certification of clinical laboratories under the Clinical Laboratory Improvement Amendments, administered by the Centers for Medicare and Medicaid Services. However, the Food and Drug Administration (FDA) has the authority to assign classifications (I, II, or III) to all medical devices and to apply increasingly stringent standards for their regulation. Historically, the implicit endorsement of a technology under FDA regulation has led to drastic consequences on uptake; for example, it was only following FDA approval of alpha-fetoprotein screening of maternal serum that American Congress of Obstetricians and Gynecologists issued its legal position that essentially assigned this novel technology as the universal standard of care [49]. A public determination has yet to be made regarding the classification of cffDNA testing for aneuploidy. Given public hearings and the sending of cease-and-desist letters to a number of companies offering genetic tests, including one company offering cffDNA testing for RhD blood type, the FDA may intend to regulate this technology as a medical device in the future [62,63]. Commercially available medical devices require pre-market notification or pre-market approval from the FDA and in response, at least one firm developing cffDNA technology has indicated that it intends to file for pre-market approval of its trisomy 21 test [64]. Given the current uncertainties of the regulatory environment, these government agencies have the ability to dictate the features and course of implementation of aneuploidy testing using cffDNA.

### Social context

As with the introduction of other prenatal technologies, campaigns that endorse or oppose the use of cffDNA testing for aneuploidy are likely to originate among activist communities, such as the disability rights community (Figure 6) [65,66]. In particular, advocacy groups for specific genetic conditions, such as trisomy 21, raise significant concerns about the underlying suggestion of prenatal testing that disability is something to be avoided and suggest that, while prenatal testing in and of itself may be acceptable, discussions surrounding prenatal options must be comprehensive, sensitive, and without undue pressures on prospective parents. Another group with considerable interest in this technology includes individuals who oppose abortion and strongly disagree with any prenatal technology that could lead to increased rates of pregnancy termination. Equally relevant groups are feminist communities or other groups in civil society that campaign for reproductive liberties, who may argue that pregnant women have an inherent right to obtain fetal genetic information via prenatal testing [67].

The influences of activism in multiple directions may manifest in state legislation over the scope of lawful prenatal testing and pregnancy termination; current legislative trends in the US include increasing restrictions on abortion [68]. In the past, some of these communities have had considerable impact on the course of research and translation of genetic technologies, through lobbying efforts, provision of research funding, and educational activities for the public and healthcare providers [67,70]. Moreover, litigation brought by private citizens, such as the aforementioned cases involving DTC cffDNA testing for fetal sex or the validity of patents on *BRCA *genetic testing, may allow the broader public to redirect future commercialization activities and uptake of these technologies [40,41,54]. As with all realms, a balance must be struck between allowing interest groups, including those whose voices are often neglected, to be heard and preventing any one constituency from wielding power to introduce genetic technologies that are ineffective or undesirable at a societal level.

At the individual level, these social and political activities may have sufficient influence to alter opinions, and thus uptake, of this technology. To the individual, the debate over whether prenatal testing or termination of pregnancy for aneuploidy is morally acceptable and the appropriate use of such information is rarely as dichotomized as it may appear on the political stage. The application of personal values to novel reproductive choices is not clear cut; pregnant women draw from intersecting and opposing messages from their healthcare providers, family, religion, culture, and society in facing such decisions [50-53]. Furthermore, families may seek prenatal test information for many reasons other than deciding whether to terminate a pregnancy; it has been an unfortunate consequence that prenatal testing technologies are often only thought of merely as precursors to selective abortions. In the related history of the implementation of maternal serum screening for aneuploidy, pregnant women often left discussions with their physicians concerned that a positive screen or test result was assumed to be cause for termination of the pregnancy. Patients ended up being unclear about the purposes or implications of screening given either their doctors' uncertainty about testing features or the lack of comprehensive, clear, and culturally sensitive communication and counseling before screening, particularly in the context of their already-complex decision-making processes surrounding uptake of this screening [52,71]. Given the parallels between cffDNA testing and this previous technology, we suggest that mechanisms be put in place to encourage careful and complete discussions among providers and prospective parents.

Ascertaining the attitudes of specific activist groups and broader sets of stakeholders, including the general public, and, to the greatest extent possible, aligning their objectives within proposed translational pathways is critical to the successful implementation of this technology. In addition, aggregate social attitudes towards cffDNA testing may influence uptake; if this testing is perceived as presenting new options to prospective parents, it is also possible that parents will be perceived as irresponsible for not availing themselves of these options [72,73]. Such attitudinal shifts may create a coercive environment for decision-making.

## Summary

Successful implementation of cffDNA testing for aneuploidy should involve the consideration of potential translational pathways and their consequences from the perspective of diverse sets of stakeholders. For example, it is critical to understand how these pathways will be influenced by the values of pregnant women, their families, and healthcare providers and mediated by the agendas of judicial and regulatory bodies, payers, professional societies, educational systems, and representatives from civil society. Throughout the translational course, researchers and developers should solicit the early and continued participation of these stakeholders in order to ensure that their visions for the ultimate use of this testing are compatible and avoid inefficient, inappropriate, or otherwise unsuccessful attempts at translation. We hope that our initial sketches of stakeholder interactions will provide a framework in which the pathways to implementation of this technology can be explored in further detail via consultation and collaboration with an array of stakeholders representing the six classes that we have described. As part of a more comprehensive study on stakeholder attitudes towards cffDNA technology, we plan to develop an interactive forum whereby diverse stakeholders, including those from traditionally underrepresented categories, can express their values and opinions and respond to the priorities of others; we urge others to develop similar methodologies for engaging stakeholders and presenting findings to those in positions to enact policies and practices that have achieved consensus among stakeholders.

### Moving forward with genomic medicine

Using cffDNA testing for aneuploidy as an example of an emerging genomic technology, we have illustrated how complex and uncertain translational pathways may be. Analyzing the priorities and dynamics among stakeholders may help to identify factors that may impact implementation and achieve a true picture of technological translation. Our assessment represents a departure from conventional technology assessment methods, which consider features inherent to the technology itself. Such assessments generally limit the inclusion of stakeholder values and lack critical historical perspectives. The situation of cffDNA testing among both genetic and reproductive technologies creates special demands on a translational analysis of this sort, requiring attention to a particularly wide scope of stakeholders and a rich history of technological, social, and political successes and failures. While the features and contextual details of other genomic technologies vary widely and must be considered on an individual basis, we argue that the successful development and provision of any new technology must be predicated on the flow of information and ideals surrounding its real and desired features among all of its stakeholders. Utilizing this framework to trace and elaborate upon translational pathways will, we believe, lead to the translation of cffDNA testing for aneuploidy in the most ethically sound manner. We hope that it may also provide a starting place for the consideration of other novel technologies - with their own unique frameworks of translational pathways - in order to achieve the goal of genomic translation in the years to come.

## Abbreviations

cffDNA: cell-free fetal DNA; DTC: direct-to-consumer; FDA: Food and Drug Administration.

## Competing interests

The authors declare that they have no competing interests.

## Authors' contributions

All authors conceived of the study and participated in its design and coordination. LCS conducted the interviews and drafted a preliminary version of the manuscript. All authors provided substantial suggestions for the manuscript and have read and approved the final manuscript for publication.
